# A Bullet in the Supraspinatus Compartment Successfully Removed by Arthroscopy: Case Report and Review of the Literature

**DOI:** 10.1155/2015/806735

**Published:** 2015-01-28

**Authors:** Alexandre Galland, Alexandre Lunebourg, Stéphane Airaudi, Renaud Gravier

**Affiliations:** ^1^Institut de la Main et du Membre Superieur (IMMS), Clinique Monticelli, Groupe Générale de Santé, 88 rue du Commandant Rolland, 13008 Marseille, France; ^2^Institut du Mouvement et de l'Appareil Locomoteur (IML), Université Aix-Marseille, Hôpital de Sainte-Marguerite, 270 boulevard Sainte-Marguerite, 13009 Marseille, France

## Abstract

Arthroscopic removal of bullet from intra-articular compartment has been described for several joints. Only few reports dealing with this condition in the shoulder have been reported especially for the glenohumeral and the subacromial compartments. We report the story of a fifty-seven-year-old man presenting a bullet in the supraspinatus compartment of his left shoulder successfully removed by arthroscopy.

## 1. Introduction

Arthroscopic removal of bullet from intra-articular compartment has been described for several joints [1], but there are only few reports dealing with this condition in the shoulder [2–4]. The use of arthroscopy in case of extraction of foreign bodies is reported to be less invasive and allows associated procedure compared to open surgery [1, 3, 4]. As our best knowledge no description of extraction of a bullet by arthroscopy from supraspinatus compartment of the shoulder has been published. We report the case of a fifty-seven-year-old man presenting a bullet in the supraspinatus compartment of his left shoulder successfully removed by arthroscopy.

## 2. Case Report

**Figure 1:**
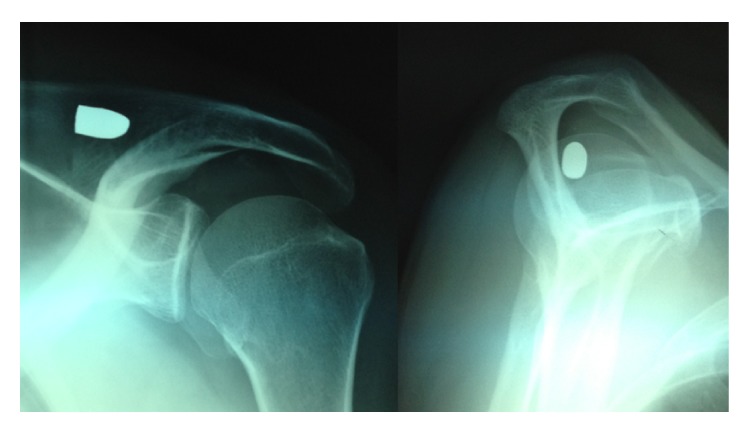
Preoperative anteroposterior and axial radiographs showing the bullet in the supraspinatus compartment of the shoulder.

**Figure 2:**
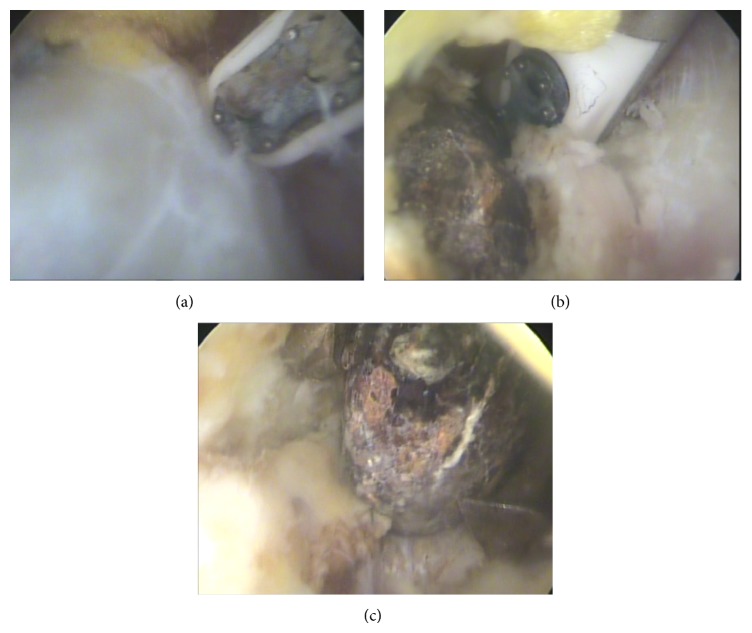
Sequence of events of the bullet extraction: (a) dissection around the bullet which is encapsulated in a fibrous scar, (b) removing of the fibrous scar around the bullet, and (c) bullet extraction with a pincer to grasp.

**Figure 3:**
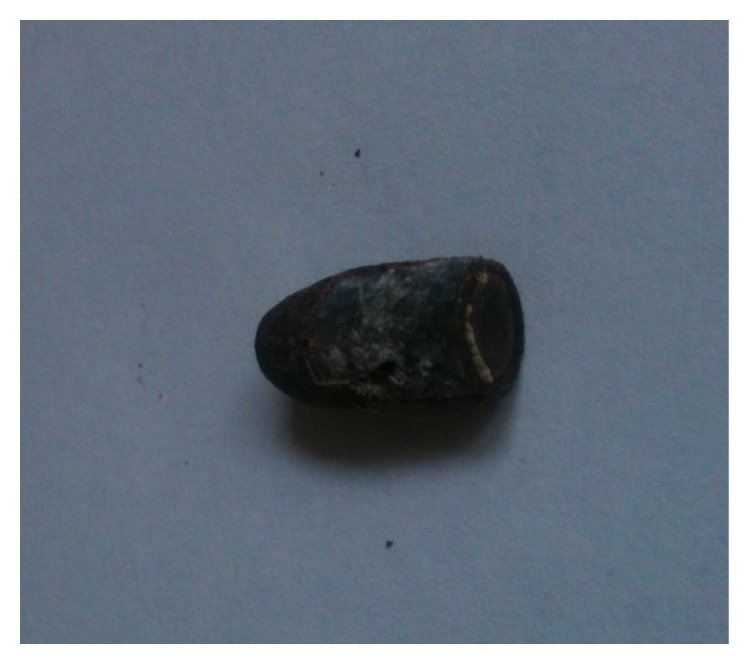
The bullet after its extraction.

A right-handed fifty-seven-year-old man in general good health complained for pain on his left shoulder for several months without limitations. In his past history, when he was thirty-one years old, he reported a shoulder gunshot injury. The bullet had a parallel course, fired from two meters, entered in the shoulder just above the collarbone, and stopped against the anterior aspect of scapula spine (Figure 1). No bone was broken and no neurovascular complications were reported. The wound healed without local complications. The patient has never been bothered by the bullet except psychological damage but presented later a clinical situation of a nontear tendinopathy due to subacromial impingement. A CT-scanner and an MRI identified a subacromial bursitis and no associated tear of the rotator cuff was identified. At this point, it was decided to perform an acromioplasty by arthroscopy and during the same procedure to remove the bullet from the supraspinatus compartment. Under general anesthesia, in beach chair position, we started by putting the arthroscope at the posterolateral border of the acromion to go in the subacromial space. Then we dissected the subacromial space to access the supraspinatus compartment through a second lateral instrumental portal and did a third approach just above the bullet in order to shave and to identify the scar fibrous tissue around the bullet. Care was taken to identify the suprascapular nerve. By the third approach we removed the fibrous scar with an electrosurgical cutting and coagulation device (ArthroCare Sports Medicine), fluoroscopy has not been used to find the foreign body. Finally we removed the bullet with a pincer to grasp (Figures 2 and 3). After we performed the bullet removal, we continued with a standard acromioplasty procedure with the section of coracoacromial ligament at its acromial insertion. No complication was encountered during the surgery. The patient stayed one day in hospital and was allowed to move his arm without restriction. At six weeks the patient moved his arm without pain and regained his prelevel activity at three months and at two years the results were identical with a Constant score at 90 points [5].

## 3. Discussion

This case report describes the removal of a low-velocity bullet from extra-articular compartment of the shoulder by arthroscopic procedure.

Previous descriptions also published reported situation of bullet extraction straight after gunshot injury and where the bullet was located on intra-articular position or subacromial space [2–6]. The uniqueness of our case report, except the arthroscopic removal of a bullet, was the extra-articular position (the anterior aspect of scapular spine) and the relative time to ballistic trauma. Indeed, the location of the bullet in the supraspinatus compartment close to the suprascapular nerve could represent a risk of lesion to the nerve by conventional open techniques. However, arthroscopic technique in this case represented a valuable and safe method, because the bullet extraction by arthroscopy has allowed us to have a continuous check of the suprascapular nerve during the bullet extraction. More, the long period between the gunshot injury and the bullet removal (twenty-six years) did not represent a problem. Indeed, the bullet was embedded in a fibrous scar, which was relatively easy to dissect with electrosurgical cutting and coagulation device. Removal according to traditional open techniques causes larger incisions, significant blood loss with neurovascular dissection, poor local visualization, and longer recovery time as was previously reported [1, 3, 4]. More, permanent irrigation decreases the risk of infection and represents an advantage compared to open surgery [1, 3, 4].

In our practice the bullet extraction for an isolated psychological reason is not an indication for surgery especially in the absence of clinical symptoms. However this patient presented a nontear tendinopathy due to subacromial impingement justifying acromioplasty associated with acromiocoracoid ligament section. The bullet extraction has been made on request of the patient who could not bear the presence of the foreign body and of the potential risk of blood toxicity due to retained bullets described in the literature, even many years after ballistic trauma [7, 8]. Again, arthroscopy in this situation allowed us to treat subacromial impingement and to extract a foreign body without adding complications, which could be very harmful for the patient.

Thus, the use of arthroscopy in the case of foreign bodies' extraction is less invasive than conventional open techniques and allows associated procedure such as precise articular evaluation (cartilage, synovial, and tendons) and irrigation. More, the bullet removal by arthroscopic technique around the shoulder is achievable regardless the bullet position (intra or extra-articular compartment) and whichever the ballistic trauma delay is.
